# Impact of Poor Adherence to Treatment on Recurrent Disease Activity in Pemphigus Foliaceus: A Case Report

**DOI:** 10.7759/cureus.109929

**Published:** 2026-05-30

**Authors:** Luisa F Vazquez Enriquez, Kenia Dominguez Romo, Jose M Hurtado Cordova

**Affiliations:** 1 Internal Medicine, Hospital General de Zona No. 33, Instituto Mexicano del Seguro Social, Monterrey, MEX

**Keywords:** adherence, autoimmune blistering disease, case report, immunosuppressive therapy, pemphigus foliaceus, septic shock, skin infection, topical therapy

## Abstract

Pemphigus foliaceus is a rare autoimmune blistering disease characterized by superficial intraepidermal blister formation and chronic relapsing disease activity. Although systemic immunosuppressive therapy remains the cornerstone of treatment, adherence to both systemic and topical therapies may influence disease control, skin barrier integrity, and susceptibility to secondary infections.

We present the case of a 73-year-old man with a history of hypertension, type 2 diabetes mellitus, and chronic ischemic heart disease who developed pemphigus foliaceus confirmed by clinical and histopathological findings. The disease initially manifested with vesicles and flaccid bullae involving the face and chest that progressed to erosions with serohematic crusts. The patient initially received systemic corticosteroids, antihistamines, and topical therapy without adequate response, requiring hospitalization and treatment with intravenous immunoglobulin. Therapy was later adjusted to include mycophenolic acid and corticosteroid tapering, achieving partial clinical improvement.

During follow-up, the patient experienced multiple relapses associated with inconsistent adherence to topical therapy and unsupervised discontinuation of systemic corticosteroids. Despite reintroduction of immunosuppressive therapy, intermittent cutaneous activity persisted. On his final hospital admission, he developed secondary cutaneous and urinary infections caused by *Klebsiella pneumoniae* and *Enterococcus faecalis*, progressing to septic shock despite advanced medical management.

This case highlights the challenges of treatment adherence in pemphigus foliaceus and emphasizes the multifactorial nature of severe disease progression in elderly patients with multiple comorbidities. Inadequate adherence, persistent disease activity, chronic immunosuppression, skin barrier dysfunction, and secondary infections may collectively contribute to unfavorable clinical outcomes. Reinforcement of patient education, close follow-up, and careful monitoring remain essential components in the management of autoimmune blistering diseases.

## Introduction

Pemphigus foliaceus is a rare autoimmune blistering disease characterized by superficial intraepidermal blister formation caused by autoantibodies directed against desmoglein 1 [1]. Clinically, it presents with fragile blisters that rapidly evolve into erosions, crusted plaques, and erythematous lesions, predominantly affecting seborrheic areas such as the scalp, face, and trunk [1,2]. Unlike pemphigus vulgaris, mucosal involvement is typically absent because of the distribution of desmoglein 1 within the epidermis [2]. Diagnosis is usually established through clinical findings supported by histopathology and, when available, immunopathological studies.

Treatment of pemphigus foliaceus is mainly based on systemic corticosteroids and steroid-sparing therapies, including immunosuppressive agents such as mycophenolate mofetil and azathioprine, as well as biologic agents such as rituximab [3]. Adjunctive topical therapy also plays an important role in reducing local inflammation, improving skin barrier integrity, and controlling cutaneous symptoms. In elderly patients and those with multiple comorbidities, disease-related skin barrier disruption, immunosuppressive treatment, and difficulty maintaining adherence to complex therapeutic regimens may increase the risk of recurrent disease activity and secondary infections.

Poor adherence to treatment may contribute to persistent disease activity, recurrent relapses, impaired epidermal barrier function, and increased susceptibility to secondary infections. In autoimmune blistering diseases, these complications may significantly increase morbidity and negatively affect prognosis [4]. We present the case of an elderly patient with pemphigus foliaceus who developed recurrent disease activity and severe infectious complications in a multifactorial clinical context, in which challenges related to adherence to both topical and systemic therapies may have contributed to disease progression and clinical deterioration.

## Case presentation

A 73-year-old man with a history of hypertension, type 2 diabetes mellitus, and chronic ischemic heart disease presented with a one-year history of an autoimmune blistering dermatosis consistent with pemphigus foliaceus based on clinical and histopathological findings. The disease initially manifested with vesicles and flaccid bullae involving the face and chest, while the mucous membranes remained spared, and progressively evolved into erosions covered by serohematic crusts.

**Figure 1:**
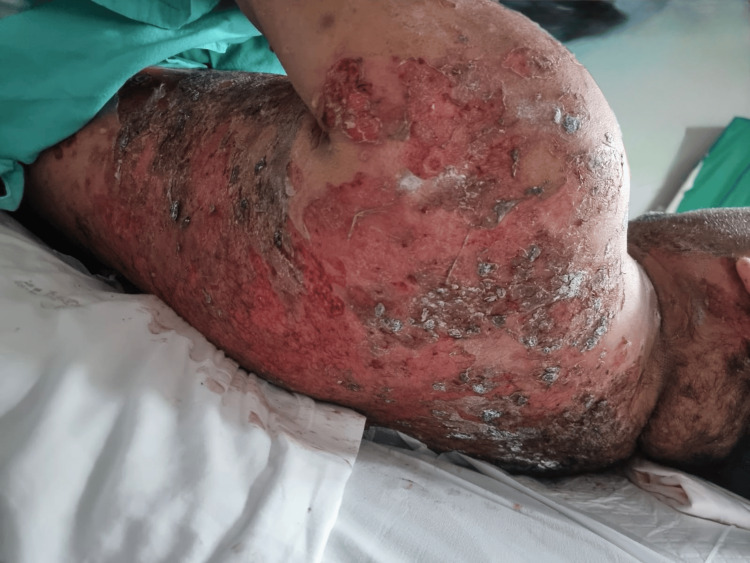
Clinical presentation of pemphigus foliaceus over the back Upper and mid back showing multiple confluent hyperpigmented and hypopigmented residual macules associated with ill-defined erythematous plaques, serohematic crusts, and superficial erosions in different stages of evolution. The lesions are consistent with active pemphigus foliaceus with residual postinflammatory pigmentary changes.

**Figure 2:**
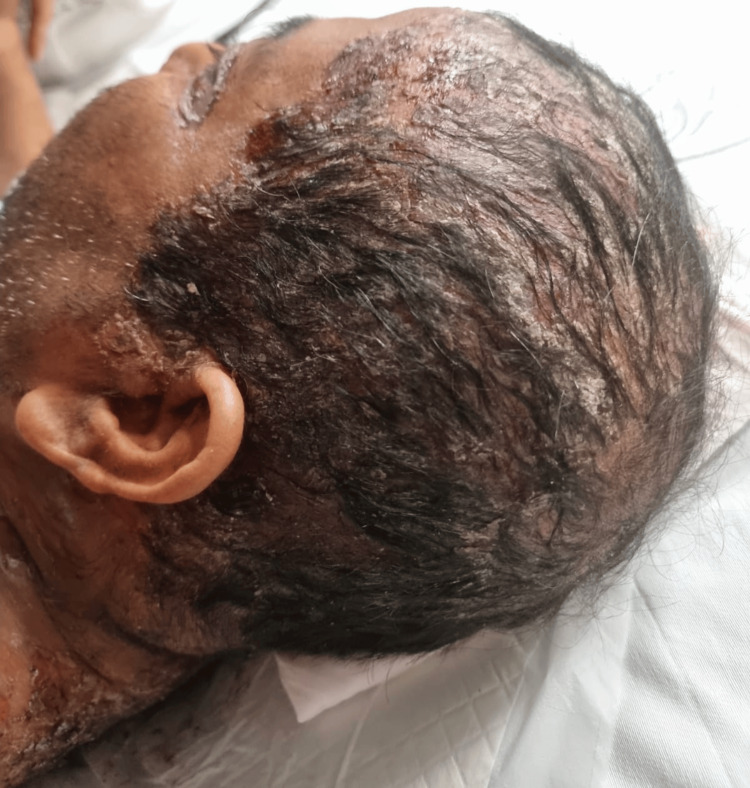
Scalp and facial involvement in pemphigus foliaceus Scalp and upper facial region showing erythematous scaly plaques covered by honey-colored and serohematic crusts, consistent with active cutaneous involvement in pemphigus foliaceus.

**Figure 3:**
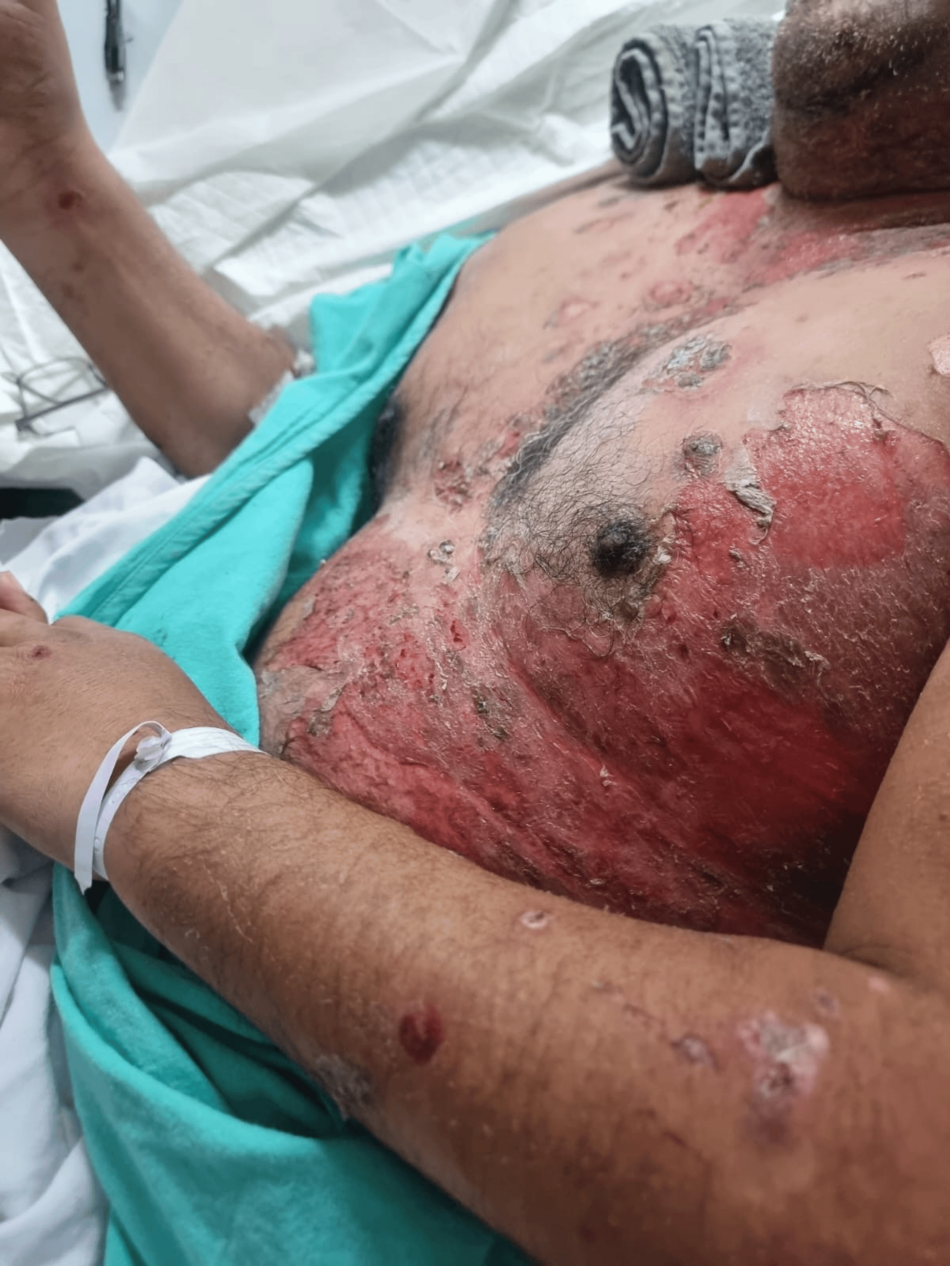
Anterior chest involvement in pemphigus foliaceus Anterior chest showing disseminated lesions in different stages of evolution, including erythematous macules, superficial erosions, and serohematic crusts, associated with active pemphigus foliaceus.

Physical examination revealed widespread cutaneous involvement affecting approximately 35% of the body surface area, with lesions in different stages of evolution. The upper and mid-back showed multiple confluent hyperpigmented and hypopigmented residual macules associated with ill-defined erythematous plaques, serohematic crusts, and superficial erosions in different stages of evolution (Figure 1). The scalp and upper facial region demonstrated erythematous scaly plaques with honey-colored and serohematic crusts (Figure 2). In the anterior chest, disseminated erythematous macules, superficial erosions, and crusted lesions were observed (Figure 3).

**Figure 4:**
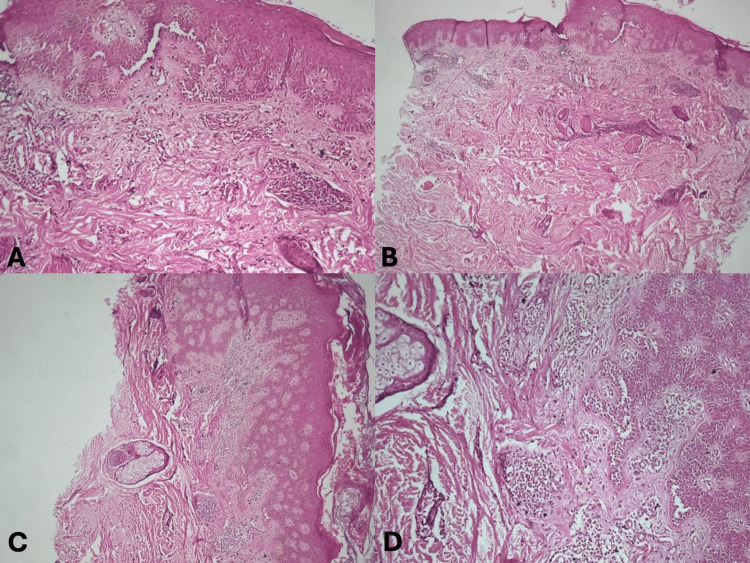
Histopathological findings (A-B) Histopathological sections showing superficial intraepidermal blister formation with subcorneal acantholysis. (C) Preservation of the basal layer attached to the basement membrane without evidence of suprabasal separation. (D) Mild spongiosis and superficial perivascular lymphocytic inflammatory infiltrate within the papillary dermis. Overall, the findings are consistent with pemphigus foliaceus.

Histopathological examination demonstrated superficial intraepidermal blister formation with histopathological features consistent with subcorneal acantholysis (Figure 4A-4B). Additional sections showed preservation of the basal layer attached to the basement membrane without evidence of suprabasal separation (Figure 4C). Mild spongiosis and inflammatory exocytosis were identified in the adjacent epidermis. In the papillary dermis, a superficial perivascular inflammatory infiltrate predominantly composed of lymphocytes was observed (Figure 4D). Direct immunofluorescence and desmoglein 1/desmoglein 3 antibody testing were not performed because of limited resource availability. However, the diagnosis of pemphigus foliaceus was supported by the clinical presentation, absence of mucosal involvement, and histopathological findings demonstrating superficial intraepidermal acantholysis.

The patient was initially treated for approximately one year with systemic corticosteroids and antihistamines, although treatment adherence was inconsistent, according to the patient. The therapeutic regimen included prednisone 50 mg daily, topical hydrocortisone 1% cream applied irregularly twice daily to affected skin areas, colloidal baths applied every eight hours to skin lesions, topical pimecrolimus once daily to facial lesions, and loratadine 10 mg orally as needed for pruritus. Due to inadequate clinical response, hospitalization was required, and the patient subsequently received intravenous immunoglobulin (IVIG) at a total dose of 190 g administered over five days, consisting of 40 g daily during the first three days followed by 35 g daily during the subsequent two days. Treatment was further adjusted to include prednisone 60 mg daily and mycophenolic acid 1,000 mg three times daily.

During follow-up, the patient initially showed clinical improvement with a reduction in disease activity. According to the patient, medications were frequently discontinued and restarted only during periods of disease flare or appearance of new lesions. Poor adherence particularly affected the prescribed topical regimen, which included hydrocortisone, colloidal baths applied to affected skin areas, and topical pimecrolimus for facial lesions. The patient also reported unsupervised discontinuation of systemic corticosteroids during the disease course. Although poor adherence to topical therapy may have contributed to recurrent flares, intermittent cutaneous activity likely reflected multifactorial disease progression despite reintroduction of immunosuppressive therapy.

On his final hospital admission, the patient developed secondary cutaneous and urinary tract infections. Urine cultures were positive for *Klebsiella pneumoniae* and *Enterococcus faecalis*. His condition progressively deteriorated, evolving into septic shock with unfavorable clinical progression despite advanced supportive management.

## Discussion

Pemphigus foliaceus is an autoimmune blistering disease caused by autoantibodies directed against desmoglein 1, resulting in loss of keratinocyte adhesion and superficial intraepidermal blister formation [1,2]. Clinically, patients develop fragile blisters that rapidly evolve into erosions and crusted lesions, predominantly involving seborrheic areas. Similar clinical manifestations have been consistently described in previous studies and remain characteristic features of the disease spectrum [5,6].

Although systemic corticosteroids and immunosuppressive agents remain the cornerstone of treatment, adequate disease control often requires a comprehensive therapeutic approach that includes adjunctive topical therapy [3]. International expert recommendations emphasize the importance of individualized long-term management strategies aimed at reducing disease activity while minimizing treatment-related complications [3,5]. Topical corticosteroids may contribute to local inflammation control, symptomatic improvement, and restoration of skin barrier integrity, particularly during periods of persistent cutaneous activity.

This case highlights the possible clinical impact of poor adherence to topical treatment in a patient with pemphigus foliaceus. Despite initial improvement after systemic immunosuppressive therapy, the patient experienced multiple relapses associated with inadequate adherence to topical management and unsupervised discontinuation of corticosteroids. Similar observations have been reported in chronic inflammatory dermatologic diseases such as psoriasis, atopic dermatitis, and acne, where poor adherence has been associated with increased disease activity, recurrent flares, prolonged treatment courses, and impaired quality of life [7-9].

Persistent skin inflammation and disruption of the epidermal barrier may facilitate microbial colonization and increase susceptibility to secondary infections, particularly in elderly and immunosuppressed patients [4]. In the present case, secondary cutaneous and urinary infections caused by *K. pneumoniae* and *E. faecalis* ultimately progressed to septic shock with unfavorable clinical evolution. Previous reports have demonstrated that severe infections remain an important cause of morbidity and mortality in patients with pemphigus, especially in older individuals with multiple comorbidities receiving immunosuppressive therapy [4,6].

In addition, the patient had multiple comorbidities, including diabetes mellitus and chronic cardiovascular disease, which likely contributed to increased vulnerability to infectious complications and poor clinical outcomes. Reinforcement of adherence strategies, patient education, and close multidisciplinary follow-up may therefore represent essential components of long-term management, potentially enabling earlier identification of inadequate adherence, reducing recurrent disease activity, and helping prevent severe complications requiring hospitalization.

This case emphasizes the importance of considering treatment adherence as a clinically relevant factor in pemphigus foliaceus management. Although systemic immunosuppression remains fundamental, optimization of adjunctive therapy and continuous patient counseling may improve disease control and overall prognosis in patients with chronic autoimmune blistering diseases.

## Conclusions

This case highlights the importance of treatment adherence as part of the comprehensive management of pemphigus foliaceus. Inadequate adherence may have contributed to persistent disease activity, recurrent relapses, impairment of the skin barrier, and increased susceptibility to secondary infections in the setting of a multifactorial clinical course. Additional factors likely associated with the unfavorable outcome in this patient included advanced age, diabetes mellitus, cardiovascular disease, chronic immunosuppression, active erosive skin disease, and unsupervised discontinuation of systemic corticosteroids. Reinforcement of patient education, close clinical follow-up, and careful monitoring of treatment adherence remain essential to improve disease control and potentially reduce the risk of severe complications in autoimmune blistering diseases.
